# Supplementary material for: Prevalence and factors associated with depression, anxiety and stress symptoms among construction workers in Nepal
Source: PLoS One. 2023 May 30;18(5):e0284696. doi: 10.1371/journal.pone.0284696 (PMC10228773; doi:10.1371/journal.pone.0284696)
Supplement: Attachment — Submitted filename: Comments on PLOS.docx [file pone.0284696.s001.docx]

**Comments from Reviewer:**

In the present work, the authors preformed the study on mental health of construction workers of Nepal and the topics is relevant to current context. This is a study of a neglected population and on sensitive topics, however, the manuscript needs revision before it is published in the journal. There is concerns multicollinearity issues and revision of tables of the result section. References need to be revised as par journal.

Abstract

Methods

Please explained why the study is community based cross sectional study. Is it necessary to add the process of ethical review in the abstract section?

Results

Please add adjusted OR when presenting the results section.

Introduction

Line 71-72, what does the information (International Labor Office in Nepal 2005) means?

Line 80, there are some error, please revise it.

Methods

I am not sure why the study is community based cross sectional study.

The author calculates the sample size of 284, however the author use 402 construction workers. It may create the confusion among the readers. So, it is suggested to revise this sentence.

In the statistical analysis section, it is suggested to do multicollinearity before doing the logistic regression whether to check whether there is correlation of the variables.

Results

It is suggested to use respondents or construction workers instead of participants.

In Table 1, it is suggested to write the number in the relevant section as well.

It is also suggested to merge castes into less category.

It is recommended to revise the table. Please make the logistic table showing the results of crude and adjusted odd ratio of depressive symptoms, similar table for anxiety and similar table for stress

It is suggested to revise the analysis if relevant. Please do the multivariate logistic regression analysis of the variable that was found to be significant in univariate logistic regression analysis.

Discussion

Please compare the prevalence of depression, anxiety and stress with National Mental Health Survey, Nepal 2020 that many reflect whether the prevalence is high, moderate and low.

Line 246-249, please revised the writing style

Line 265-266, please revise the writing style

Line 288-298, please review whether this sentence is importance and relevant to the manuscript.

References
Please revise the reference and follow the referencing style based on the journal requirement. In the reference section, some journals are in full name and some journals are in abbreviations.
