# Supplementary material for: Prevalence and factors associated with depression, anxiety and stress symptoms among construction workers in Nepal
Source: PLoS One. 2023 May 30;18(5):e0284696. doi: 10.1371/journal.pone.0284696 (PMC10228773; doi:10.1371/journal.pone.0284696)
Supplement: Attachment — Submitted filename: response to reviewers.docx [file pone.0284696.s002.docx]

Respected reviewers,

I would like to thank all reviewers for their wonderful feedback. The response to the reviewer’s comment are as follows:

Reviewer #1: This is a cross-sectional study conducted with construction workers of Nepal. The authors aim to assess the prevalence of depressive, anxiety and stress symptoms and their association with socio-demographic, life-style and occupational factors among construction workers of Kavre district, Nepal. The biggest limitation of the study is the convenience sampling which makes it difficult to infer current conclusions. I have a number of suggestions below:

1. I recommend the objective be rephrased as “…..to assess psychological distress (depression, anxiety and stress) and associated factors among construction workers in Kavrepalanchok district, Nepal” instead of “…..to assess the prevalence of depressive, anxiety and stress symptoms and their association with socio-demographic, life-style and occupational factors among construction workers of Nepal. ”

Answer: We have corrected as per your suggestion.

2. Please provide references for lines 83-87.

*Answer: Reference is added to the line 83-87.*

3. Methods section should be more detailed. For example, how was the study area selected, how many wards were there in each municipality and how many were targeted for data collection, and who collected the data, etc.

*Answer: the method sections are changed as per your suggestion by providing details you expected.*

4. Authors defined construction workers as members of specialist trades such as builders, electricians, carpenters, bricklayer, manual labor, armature fixing workers, internal finish workers and plumbers in the introduction. As stated in the inclusion criteria, is it only building construction workers who were interviewed in the present study? Please clarify.

*Answer: It is clarified*

5. Could the authors explain how current smokers and current drinker were defined in the study? How did the authors decide which risk factors to investigate in this study? I suggest writing a separate paragraph explaining the variables that were assessed.

*Answer: It is explained in the current version of manuscript.*

6. Throughout the manuscript, I suggest the authors use either Kavre or its full name.

*Answer: Done*


7. Please correct “convenient” to “convenience” on line 116.

*Answer: Done*

8. Could the authors explain why they sought ethical approval from B.P. Koirala Institute of Health Sciences sought rather than the NHRC?

*Answer: This manuscript is the part of thesis carried out in BPKIHS. The IRC of BPKIHS is the body of NHRC to which NHRC have given certain rights to provide ethical approval for observational studies including cross-sectional studies.*

9. Please correct “life-style” to “lifestyle” on line 135 and throughout the manuscript.

*Answer: done*

10. Authors mentioned interquartile range on line 174 but it does not appear anywhere else in the manuscript. Is there a reason to mention it?

*Answer: The comment is addressed*.

11. Results: What was the response rate?

*Answer: The response rate is added to first part of result section.*

12. The authors reported Tamang, Bhote, and Sherpa as ethnic groups on line 180 but this classification was not shown in the tables. It would be helpful if the authors could provide an explanation of how the classification of ethnicity was made and if applicable, the reference for it.

*Answer: The classification of the ethnicity is explained in the method section.*

13. Could the authors explain how was the multivariate logistic regression performed and why?

All variables from univariate analysis were kept in the multivariate logistic regression model.

*Answer: All variables in univariate logistic regression were included in multivariate logistic regression analysis.*

14. Odds ratios in univariate and multivariate analyses are not correctly interpreted. I suggest the authors revise it.

*Answer: We revised the interpretation of univariate and multivariate*

15. Could the authors provide a reference to Kobotoolbox?

*Answer: reference is added to kobotoolbox*

16. Line 273, 'Nepalease' should be changed to 'Nepalese,' and 'Vrahamin' should be changed to 'Brahmin,' as well. Line 282: please spell socioeconomic correctly.
*Answer: Changed*

17. Tables description should explain all the symbol in the table body like CI, n, COR, AOR etc.

*Answer: Added*

18. Line 234, I suggest that authors also include the digits following the decimal.

*Answer: Digits following decimal are added in line 234*

19. Discussion: What could be the future research direction after this study?

*Answer: It is added now in the manuscript*

20. Lines 303-305: please complete the sentences.

*Answer: Edited*

21. Authors should also expand the limitations and biases associated with the sampling strategy.

*Answer: A limitation related to the sampling strategy is added to the manuscript.*

22. The recommendation ‘to develop and implement evidence-based and appropriate community-based mental health prevention programs for laborers and construction workers’ needs some further explanation.

*Answer: This comment is addressed.*

23. Attaching a STROBE checklist would strengthen your study.

*Answer: Strobe guideline is followed but not attached here with this manuscript*

24. There were several grammatical errors in the manuscript. The authors should use professional editing services to edit the manuscript.

*Answer: We corrected grammatical mistakes that we found in the manuscript.*

Reviewer #2: In the present work, the authors preformed the study on mental health of construction workers of Nepal and the topics is relevant to current context. This is a study of a neglected population and on sensitive topics, however, the manuscript needs revision before it is published in the journal. There is concerns multicollinearity issues and revision of tables of the result section. References need to be revised as par journal.

*Answer: Multicollinearity testing was conducted to check for the multicollinearity issue. The variance inlation factor (VIF) was used to identify multicollinear. All variables have VIF less than 2.5. The tables are kept to match the need of journal and to prevent longer tables, regression tables are splitted to univariate and multivariate tables.*

Abstract
Methods
Please explained why the study is community based cross sectional study. Is it necessary to add the process of ethical review in the abstract section?

*Ans: The comment is addressed*

Results
Please add adjusted OR when presenting the results section.

*Ans: corrected*

Introduction
Line 71-72, what does the information (International Labor Office in Nepal 2005) means?

*Ans: this issue is resolved*

Line 80, there are some error, please revise it.

*Ans: the issue is resolved*

Methods
I am not sure why the study is community based cross sectional study.

*Ans: The study was conducted directly in the community so it’s a community based crossectional study but as per your comment and prevent confusion, only crossectional study is kept as study design*

The author calculates the sample size of 284, however the author use 402 construction workers. It may create the confusion among the readers. So, it is suggested to revise this sentence.

*Ans: The statement is revised*

In the statistical analysis section, it is suggested to do multicollinearity before doing the logistic regression whether to check whether there is correlation of the variables.

*Ans: Multicolinearlity testing was conducted before selecting variables in the model*

Results
It is suggested to use respondents or construction workers instead of participants.

In Table 1, it is suggested to write the number in the relevant section as well.
It is also suggested to merge castes into less category.

*Ans: The caste was reduced to 4 categories from larger number of categories and cant be merged because with merging and reducing categories will result in loss of information.*

*It is recommended to revise the table. Please make the logistic table showing the results of crude and adjusted odd ratio of depressive symptoms, similar table for anxiety and similar table for stress*

*With this suggestion the count of tables will increase which is not recommended as per journal requirement so we planned to keep two tables, one with univariate analysis and another with multivariate analysis.*

It is suggested to revise the analysis if relevant. Please do the multivariate logistic regression analysis of the variable that was found to be significant in univariate logistic regression analysis.

*Ans: Since it is not aimed to generate prediction model, we kept relevant variables to explore association between outcome and independent variables*

Discussion
Please compare the prevalence of depression, anxiety and stress with National Mental Health Survey, Nepal 2020 that many reflect whether the prevalence is high, moderate and low.

*Ans: The National Mental Health Survey, Nepal 2020 have not determined depression, anxiety and stress symptoms so we could not compare our findings with National Mental Health Survey, Nepal 2020.*

**Line 246-249, please revise the writing style**

*Answer: The line 246-249 is revised for clearness*

Line 265-266, please revise the writing style

*Ans: Line 265-266 is rephrased*

Line 288-298, please review whether this sentence is importance and relevant to the manuscript.

*Ans: Line 288-298 is corrected*

References
Please revise the reference and follow the referencing style based on the journal requirement. In the reference section, some journals are in full name and some journals are in abbreviations.

*Ans: All references are corrected as per the referencing style of the journal*

Thank you very much for your valuable feedback. It was very helpful to improve this manuscript.

Best Regards

Bikram Adhikari
