# Supplementary material for: Prevalence and factors associated with depression, anxiety and stress symptoms among construction workers in Nepal
Source: PLoS One. 2023 May 30;18(5):e0284696. doi: 10.1371/journal.pone.0284696 (PMC10228773; doi:10.1371/journal.pone.0284696)
Supplement: Attachment — Submitted filename: Response to reviewers.docx [file pone.0284696.s003.docx]

Thank you to all reviewers and editors for providing constructive feedback and comments. I’ve addressed all comments that you have provided in the manuscript and submitted it again after careful revision of grammar and errors.

Reviewer #1: I appreciate your thorough responses to reviewer questions and concerns. In my previous comment, I suggested that authors rephrase the objective as “…..to assess psychological distress (depression, anxiety and stress) and associated factors among construction workers in Kavre district, Nepal”. The authors stated in their response that they have changed as per the suggestion; however, Kavre has not been mentioned in the objectives of the abstract and introduction. Results, 41.3% is not a majority, revise the wording. Besides these points, the authors have addressed my comments.

**Ans: Thank you reviewer 1, I’ve addressed this comment in the new submitted manuscript.**

Reviewer #3: This is a cross-sectional study conducted among 402 construction workers in Banepa and Panauti municipalities of Kavre district, Nepal aimed to assess psychological distress (depression, anxiety and stress symptoms) and its association with socio-demographic, lifestyle, and occupational factors among construction workers in Nepal. I have mentioned few suggestions below:
Abstract
1. Introduction: In line 35, i suggest authors to use aimed instead of aims.

**Ans: Corrected**
2. Introduction: From line 86-94, same reference 6 has been used by the author. I recommend author to add any other reference article with similar information if its available. I suggest the authors to write the objectives as written in the abstract section: to assess the prevalence of depression, anxiety and stress symptoms and its association with socio-demographic, lifestyle and occupational factors among construction workers in Nepal.

**Ans: Corrected**

3. Methods: I recommend authors to mention the study as cross-sectional study. community based is not required to be mentioned.

**Ans: Corrected**

4. Data collection: Could the authors explain regarding translation of english questionnaire into the local language and back-translation into English. I would suggest to provide information regarding pre-testing if it was performed. In line 148, 'b' is to be added in 'uddhism'.

**Ans: Corrected**

5. Line 177, mention as 'depression, anxiety, and stress symptoms'.

**Ans: Corrected**

6. In line 196, for statistical analysis authors mentioned use of 'SPSS version 20' whereas in abstract section in line 45, its mentioned 'R version 3.6.2'. Please clarify.

**It was a typo, We used R version 3.6.2 for statistical analysis. Thank you addressing this serious issue**

7. Line 217, 61.25% is missing.
8. In Univariate and Multivariate analysis table, what does 1 denote? You may also signify it as Ref. Mention at the end of table, Ref: Reference group if relevant.

**Ans: Changed and annotated**

9. Line 221, its better to use 'depression' than 'depressive'.

**Ans: Corrected**

10. In description of Table 2, authors should also add regarding ethnicity and stress symptoms as it is significant as shown in table.

**Ans: Corrected**

11. Tables description should explain only the symbol used in the table body like CI, COR or AOR etc. Authors can also mention Title of the table and in bracket total study population as (N=402).

**Ans: Corrected**

12. There are grammatical errors in the manuscript. The authors are requested to go through it and edit the manuscript where required.

**Ans: Corrected**

13. I suggest authors to revise the references as per the referencing style based on the journal. Also fonts of the text should be similar. Please consider.

**Ans: Corrected**
